# Nanoparticle suspensions enclosed in methylcellulose: a new approach for quantifying nanoparticles in transmission electron microscopy

**DOI:** 10.1038/srep25275

**Published:** 2016-05-04

**Authors:** Christian Hacker, Jalal Asadi, Christos Pliotas, Sophie Ferguson, Lee Sherry, Phedra Marius, Javier Tello, David Jackson, James Naismith, John Milton Lucocq

**Affiliations:** 1 School of Medicine, University of St Andrews, St Andrews, Fife, UK; 2 Centre for Biomolecular Sciences, University of St Andrews, St. Andrews, Scotland; 3 Biomedical Sciences Research Complex, University of St Andrews, North Haugh, St Andrews, United Kingdom

## Abstract

Nanoparticles are of increasing importance in biomedicine but quantification is problematic because current methods depend on indirect measurements at low resolution. Here we describe a new high-resolution method for measuring and quantifying nanoparticles in suspension. It involves premixing nanoparticles in a hydrophilic support medium (methylcellulose) before introducing heavy metal stains for visualization in small air-dried droplets by transmission electron microscopy (TEM). The use of methylcellulose avoids artifacts of conventional negative stain-TEM by (1) restricting interactions between the nanoparticles, (2) inhibiting binding to the specimen support films and (3) reducing compression after drying. Methylcellulose embedment provides effective electron imaging of liposomes, nanodiscs and viruses as well as comprehensive visualization of nanoparticle populations in droplets of known size. These qualities facilitate unbiased sampling, rapid size measurement and estimation of nanoparticle numbers by means of ratio counting using a colloidal gold calibrant. Specimen preparation and quantification take minutes and require a few microliters of sample using only basic laboratory equipment and a standard TEM.

Nanoparticles are of growing importance in biology, medicine, pharmacology, cosmetics and environmental studies. Indeed a range of synthetic nanoparticles are used increasingly as drug carriers^1^,^2^, diagnostic and analytic tools^3^, therapeutic reagents^4^,^5^,^6^,^7^, or as platforms for membrane proteins in structural and functional studies^8^,^9^. The materials used for synthesis can be in “condensed” form, as in metallic colloids or carbon nanotubes or composed of “soft” biological substances such as the lipids used to make liposomes, nanodiscs or bicelles. Nanoparticles can also occur naturally with those of particular significance being pathogenic viruses, important for diagnosis or vaccine development, or the extracellular vesicles called exosomes that are emerging as markers of human disease^10^. In many of these applications quantitative monitoring of nanoparticles is essential and demands rapid, accurate and sensitive quantification on the smallest possible samples at high-resolution. The primary morphological parameters of interest are particle number (concentration), particle size and the degree of aggregation^11^.

Currently nanoparticle quantification is based mainly on biophysical approaches^12^. These include nanoparticle-tracking analysis (NTA)^12^,^13^,^14^, flow cytometry^15^, dynamic light scattering (DLS or photon correlation microscopy)^16^,^17^, small angle X ray analysis and resistive pulse sensing. However these methods are largely indirect in nature and can be prone to systematic errors, especially when nanoparticles vary in size or aggregation state. A more direct approach for characterizing nanoparticles is electron microscopy^11^, which provides nanometer-scale visualization of particles and their morphology. Current EM technologies suited to nanoparticle investigation include TEM, focused ion beam scanning EM (FIBSEM) and scanning EM (SEM)^11^. In particular section based methods such as TEM and FIBSEM are especially powerful when applied to nanoparticles that are dispersed in solid matrices where estimating particle numbers and matrix volume are relatively straightforward and the aggregation state is well preserved. However a significant problem occurs when characterizing nanoparticles in liquid phase for which there are a number of possible solutions. The first is to visualize the dispersion directly in the liquid state, involving encapsulation in specialized containers that can be inserted into the vacuum needed for electron microscopes to function^11^. However, this technology is still at an early stage and there are concerns about effects of the electron beam on the nanoparticles. Another solution is to capture or solidify the nanoparticle suspension by rapid freezing and visualize in the frozen state – a technique referred to as cryo-EM. Advantages of this approach include preservation of aggregation state and hydration^18^, as well as a lack of a requirement for heavy metal contrast. Therefore cryo-EM represents an optimum approach for qualitative observation^19^ although quantification remains technically challenging because variations in specimen quality make proper exhaustive analysis or proper sampling difficult. A third solution for visualizing nanoparticles that are dispersed in liquid phase is to dry the particle suspension in such a way as to avoid morphological or chemical modification through denaturation, and to avoid artifactual aggregation.

A classical technique for structural analysis that uses drying of nanoparticle suspensions is negative stain TEM^20^. Here nanoparticles are absorbed onto an EM support and heavy metal stain applied. This method is the mainstay of high resolution analysis revealing structural details of soft nanoparticles (lipid membranes and proteins) as well as nanoparticles composed of condensed, “hard”, materials such as metallic particles, carbon or plastics. Importantly negative stain has not come into general use for quantifying nanoparticles, because it can generate artifacts such as particle collapse, aggregation, and non-uniform deposition^20^. A key problem is that the adsorption of nanoparticles to the EM support film cannot be easily controlled, resulting in a poor representation of the whole particle population for sampling. Decades ago, attempts to overcome this problem used calibration beads that were applied to EM grids by centrifugation, spraying or drying along with the nanoparticles^21^,^22^,^23^,^24^,^25^,^26^; but the results had low precision, were lengthy and relied on quantitative assumptions about the bead preparations. In the interim TEM has been viewed as slow and technically demanding, producing results of variable quality^15^.

With these problems in mind we developed a simple TEM method that uses embedment of aqueous suspensions of nanoparticles within hydrophilic films composed of methylcellulose. Distinct from the negative stain approach, the method disperses nanoparticles in standard volumes of methylcellulose containing uranyl acetate stain before drying. The method restricts aggregation and patchy distributions, and hinders particle collapse while retaining fine structure. Comprehensive visualization of the nanoparticle population allows unbiased sampling for measurement and characterization of a range of nanoparticle types. Methylcellulose also allows stabilization of metal calibration particles in the hydrophilic films, facilitating estimation of nanoparticle numbers/concentration with precision and sensitivity. Preparation and quantification protocols are achieved in minutes and require only standard laboratory and imaging equipment.

## Results and Discussion

### Methylcellulose films

**Figure 1:**
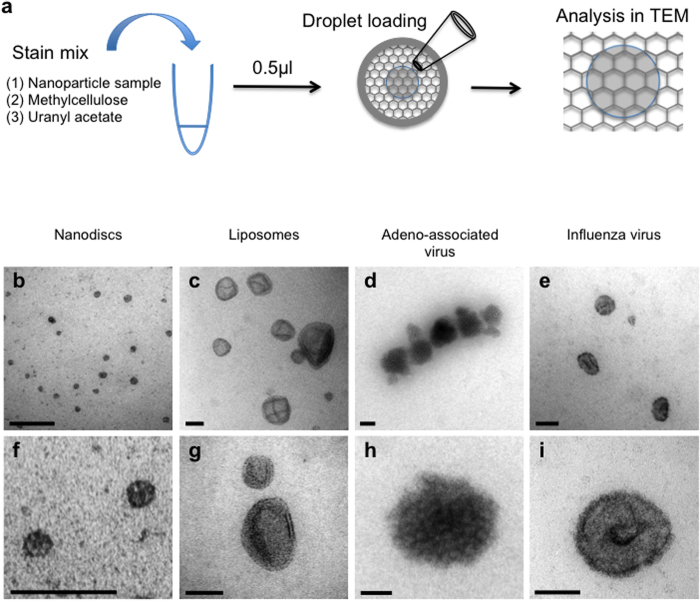
Nanoparticles in methylcellulose films imaged in TEM. (**a**) The stain mix contained 1.5 μl of 1% methylcellulose, 7.5 μl of nanoparticle suspension and 1 μl of 0.3% uranyl acetate (uranyl acetate was added after dialysis if required; see text and Fig. 3). 0.5 μl was loaded onto a standard pioloform coated EM grid support and allowed to dry before examination in the TEM. (**b**–**i**) A range of nanoparticles imaged at low magnification (**b**–**e**) and high magnification (**f**–**i**). Scale bars (**b**–**e**) 100 nm and (**f**–**i**) 50 nm.

Previously methylcellulose films containing electron dense stains have been used extensively for embedding ultrathin cryosections^27^,^28^,^29^ and also for visualizing isolated vesicular organelles but only after their adsorption to EM supports^30^. Our aim was to test whether methylcellulose could stabilize suspensions of nanoparticles, so that standardized volumes of hydrophilic films could be mounted for studying and sampling the whole nanoparticle population in the TEM. Nanoparticle suspensions were first mixed with a solution of methylcellulose before adding a heavy metal contrasting agent (uranyl acetate). The mixture was then applied to the center of a support plastic-coated TEM grid as a 0.5 μl droplet containing 0.15% methylcellulose and 0.03% uranyl acetate (Fig. 1a). Once dried, the film formed a circular spot, which was thicker near the rim. Such a “coffee ring” distribution is a common effect observed during drying of fluid droplets^31^,^32^. Importantly we found that structural display of liposomes, viruses and nanodiscs (Fig. 1b–i) was consistent across the whole droplet even in the thicker peripheral parts of the droplet and most nanoparticle preparations tested remained aggregate free. We found larger droplets (i.e. >0.5 μl) dried to form regions of high electron density, obscuring some particles, while smaller volumes produced films with patchy contrast and marked “collapse” of vesicular structures. Visualizing all nanoparticles in the droplet was an important prerequisite for unbiased sampling, accurate measurement and enumeration of the particles in the droplet (described later).

Interestingly, the uranyl acetate concentration used was much more dilute than that commonly used for cryosection contrasting in methylcellulose^27^,^28^,^29^. The staining mechanism likely involves local adsorption of the heavy metal onto nanoparticles rather than conventional “negative” staining in which biological structures are highlighted by surrounding lakes of stain. This “positive” staining of nanoparticles could provide novel structural information, especially if combined with tomographic analysis of particle structure.

**Figure 2:**
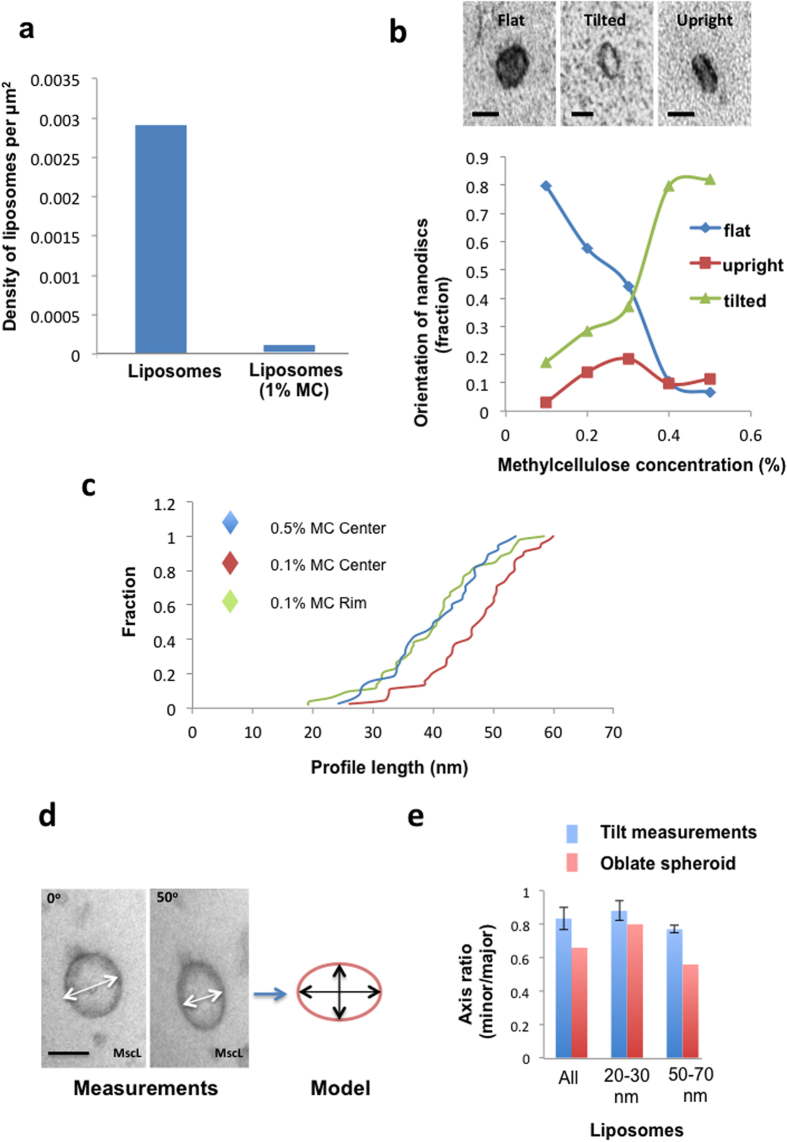
Methylcellulose restricts binding to the substratum, allows freedom of orientation and prevents substantial collapse of nanoparticles. (**a**) Binding to substratum. Liposomes were incubated in 1% methylcellulose or water before adsorbing the mix to an EM grid, contrasting, and density of liposomes determined. (**b**) Orientation. 12 nm diameter nanodiscs were mixed with methylcellulose solution varying between 0.1% or 0.5% before adding uranyl acetate. 0.5 μl droplets were loaded onto EM grids and dried before classifying orientation by TEM (flat, tilted and upright). Nanodiscs become progressively tilted with increasing concentration of methyl cellulose. Bars, 7nm. (**c**) Gold nanorods were mixed with 0.1% or 0.5% MC. 0.5 μl drops were loaded onto EM grids and nanorods situated in either the rim or center were measured. Data are presented as cumulative fractions. Nanorods in the thinnest films (0.1% MC center) were longer compared to those in thicker films at the 0.1% MC rim (Kolmogorov-Smirnov test, P 0.001; n 45 and 52 data points respectively) or the 0.5% MC center (Kolmogorov-Smirnov test, P 0.01; n 45 and 38 data points respectively). The data are consistent with increased freedom to rotate in thicker MC films. (**d**,**e**) Tilt analysis of vesicle compression in methylcellulose films. Vesicles containing (MscL) pore protein were embedded in methyl cellulose as described in the text and the degree of flattening (oblate spheroid; model) estimated from minor and major axes measured after 50° specimen tilt. Data are from the whole population (All), n = 38, 20–30 nm vesicles, n = 16 and 50–70 nm vesicles, n = 10. Bar = 50 nm.

Nanoparticle suspensions dried in droplets of methylcellulose/uranyl acetate appeared largely free of aggregates, which indicated that methylcellulose prevented interactions by adhering to particle surfaces as an inert coating^33^. This was further tested using colloidal gold particles, which are well known to aggregate in the presence of cations. When colloidal gold nanoparticles were added to 1% methylcellulose and phosphate buffered saline added, the gold particles failed to aggregate substantially (as indicated by the lack of a color change from red to blue/black). In addition this screening effect appeared to reduce binding of nanoparticles to the plastic EM grid support film as evidenced by the strong inhibition of liposome binding to the grid support (Fig. 2a).

The possible mechanisms by which MC prevents particle-particle and particle surface interactions in solution and during drying are of interest. There is a large literature showing that polymers bind to metallic or plastic surfaces via short-range forces (van der Waals), with longer polymers binding tighter than shorter ones (reviewed in ref. 34). In the standard model of particle-particle interactions, particle surface charges restrict aggregation by electrostatic repulsion, but on addition of electrolytes the surface charges can be neutralized resulting in aggregation. Importantly when hydrophilic amphiphilic polymers are bound to the particles, the resulting screening effect allows the nanoparticles to take on properties of the polymer and thereby decreasing the impact of particle attraction and reducing salt-induced aggregation^34^. Methylcellulose is a neutral water-soluble cellulose derivative produced by heterogeneous methylation of semi-crystalline cellulose. Previous work demonstrating adsorption of methylcellulose to silica particles surfaces supports this view^33^. It is therefore likely that MC coats the surfaces involved in TEM processing, preventing particle aggregation as well as adsorption/distortion at the grid support surface. It appears that MC acts in this way throughout the specimen preparation and drying processes, until the MC becomes solid.

In conventional negative stain, particles adhere to the grid support and assume preferred orientations after drying. We argued that nanoparticles embedded in the methylcellulose droplet as a suspension should now demonstrate freedom to orientate during drying. To test this we prepared nanodiscs that were 12 nm in diameter^35^ and embedded them in methylcellulose films of varying final thickness before assessing their orientations. Nanodiscs are synthetic lipoprotein nanoparticles used as carriers for membrane proteins. In the thinnest films (made with 0.1% methylcellulose) most of the nanodiscs were oriented “enface” to support film after drying (Fig. 2b). However in thicker films (made with 0.3–0.5% methylcellulose), a range of tilted orientations became apparent, showing nanodiscs were increasingly free to orientate during the drying process with increasing film thickness. To further test whether other asymmetric nanoparticles assume multiple orientations in MC droplets, we embedded gold nanorods in films of varying thickness and measured their projected profile lengths in TEM images (Fig. 2c). In the thinnest central regions of droplets, made with 0.1% MC, nanorod profiles were on average longer (mean 46.59 nm coefficient of error 2.5%, n = 45) than those measured in the thicker rim in 0.1% MC droplets (mean 39.87 nm coefficient of error 3.1%, n = 52) or the central region of film made with 0.5% MC (mean 36.6 nm coefficient of error 3.2%, n = 38). Tilting analysis in TEM confirmed that shorter rod profiles found in thicker films were at orientations tilted relative to the horizontal (not shown); by comparison nanorods in thinner parts of the film lay uniformly horizontal to the MC film (statistical comparison of nanorod lengths are shown in the legend to Fig. 2c). We also obtained evidence that nanoparticle suspensions are distributed throughout the thickness of dried MC droplets. After drying droplets containing mixes of gold particle calibrant and MC, gold particles in the droplet rim appeared to form clusters/aggregates. However these clusters could be clearly separated by tilting the EM grid relative to the electron beam demonstrating that the nanoparticles spread through the thickness of the droplet film in the vertical direction.

Published data predicts significant collapse of negatively stained synaptic vesicles. For example the data of one study indicates a height to width ratio of 0.25 for 47 nm vesicles^36^. We next tested for collapse of nanoparticles suspended in methylcellulose using liposomes that had spherical morphology. These liposomes carried the protein channel MscL^35^, and as previously shown for the mechanosensitive ion channel MscS^35^,^37^,^38^,^39^, it allows for the import of water into the vesicles making them appear turgid and pseudo-spherical. The degree of flattening after drying was evaluated by comparing images at 0 and 50° tilt, with consequent modeling of vesicles as oblate spheroids. MscL vesicles had an aspect ratio (height to width) of 0.66 after drying. Overall at this level of compression the volume equivalent vesicle sphere had a diameter, which was 0.88 of the spheroid major axis measurement indicating an overestimation of vesicle size of approximately 12% using the methylcellulose method. Interestingly larger turgid vesicles appeared more flattened (50–70 nm diameter (26% of the population); aspect ratio 0.56) than smaller ones (20–30 nm (34% of the population); aspect ratio 0.8; Fig. 2c). We conclude that methylcellulose suspensions support vesicles better than negative stain. Another interesting approach to reduce collapse of structures could be cryo-EM, but this technique was not investigated further because, as with negative stain, visualization of particle populations is patchy making valid sampling and quantitation problematic^40^.

### De-salting nanoparticle preparations using a novel microdialysis system

**Figure 3:**
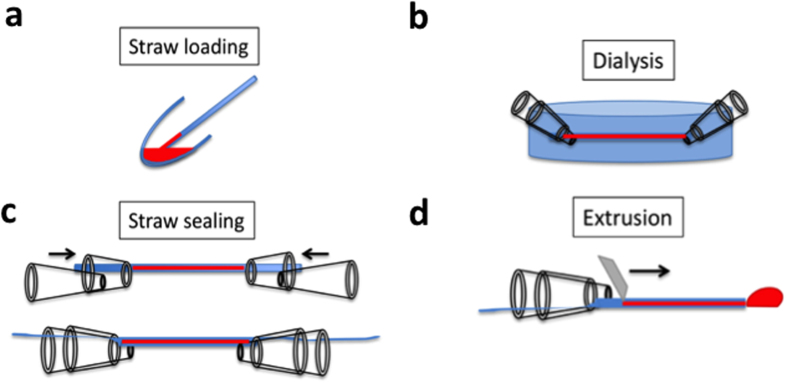
Microdialysis procedure using semipermeable cellulose kidney dialysis capillaries. (**a**) Three to five μl of sample was drawn into the kidney dialysis straw by capillary action. (**b**) Ends of the straw were sealed using an assembly made from interlocking truncated micropipette tips. (**c**) The assembly was transferred into the dialysate. (**d**) The dialyzed sample was extruded onto parafilm and added to methylcellulose/uranyl acetate mix before loading and drying on an EM support grid.

Biological nanoparticle preparations contain salts that interfere with electron stains and/or may crystalize during drying, thereby obscuring nanoparticles. The applicability of the method was further enhanced with a microdialysis system (Fig. 3) that makes use of cellulose dialysis straws obtained from kidney dialysis units (internal diameter of the capillaries was approximately 200 microns with a molecular weight cut-off of 10,000 Da). A mix of methylcellulose solution and colloidal gold calibrant was combined with nanoparticle solution and three to five microliters loaded into straws by capillary action before sealing (Fig. 3a). After dialysis against deionized water, the dialysate was extruded and heavy metal stain added before loading the droplet onto an EM grid (Fig. 3b–d). After dialysis, neither aggregation of gold particles or NPs, nor precipitation of stain was observed. Importantly we did not observe blebbing of liposomes or virus under the low molarity conditions. This novel procedure allowed microliter samples containing nanoparticles to be processed for quantitation in a variety of culture supernatants, laboratory samples and body fluids.

### Quantifying nanoparticle size

**Figure 4:**
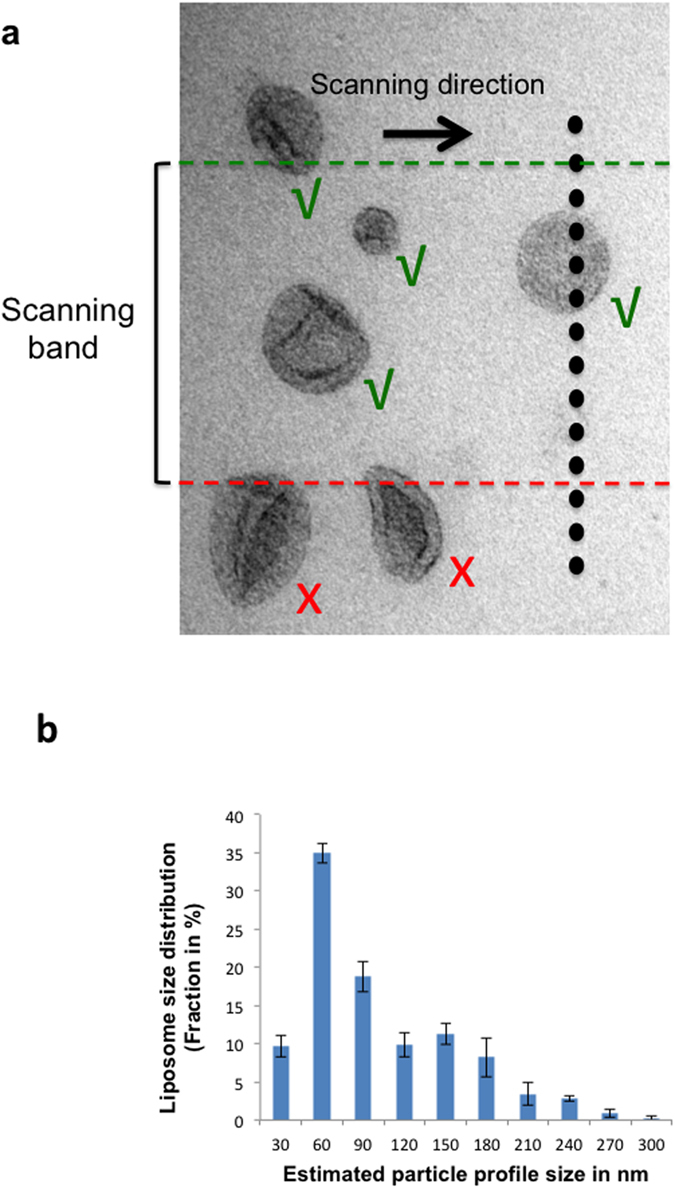
Liposome sampling and size estimation. Liposomes were sampled using a scanning band to which an unbiased counting rule was applied (**a**). All particles passing completely between the lines or intersecting the acceptance line (green) were selected for analysis. Particles intersecting the forbidden line (red) were excluded. Selected particles were translocated through size a systematic series of dots the upper edge of each tracing a scanning line. The particle caliper diameter was calculated from the number of intersection of dot edges with the nanoparticle and the real spacing of the dots as described in the text. Data are from three experimental runs from the same liposome preparation (**b**). Approximately 100 vesicles were counted per experiment; error bars are standard error of the mean (n = 3; each estimation took approximately 10 minutes).

We next developed a method for particle sizing by combining the resolution of TEM with a sampling based stereological method. To increase throughput the method was applied to a live digital camera display during x/y scanning across the grid. The principle was tested using liposomes that are used widely for drug/reagent delivery and as carriers for study of protein function^41^. Liposomes embedded in methylcellulose droplets were selected during x/y scanning by applying unbiased counting rules to a scanning-band (Fig. 4)^42^,^43^. The selected liposomes were then translocated through a systematically spaced array of dots that was placed across the digital camera display (Fig. 4a; see ref. 42). In this system the number of intercepts between each translocating liposome with the upper dot edges can be used to estimate particle caliper diameter unbiasedly (where C_d_ (caliper diameter) = I_lip_ (number of intercepts with the liposome) × d (spacing of the dot edges)); given random positioning of the particles and/or line array. For each preparation the results from approximately 100 liposomes were grouped into at least 10, 30 nm size bands (Fig. 4b). Evaluation of each liposome preparation took 10 minutes and over three experiments the average coefficient of error for size estimates over all size bands under 210 nm diameter was 8%. The distributions from the three experiments were not distinguishable as determined by contingency/Chi square analysis (p > 0.25, df 16).

### Quantifying gold calibrant

The principle was to mix gold particle calibrant (15 nm gold particles at known concentration and prepared with the citrate reduction method^34^,^42^) with nanoparticles of interest and count the ratio of gold to nanoparticles. As already noted, salt-induced aggregation of colloidal gold^34^ was restricted in 1% methylcellulose, which allowed the calibration solution to be premixed with nanoparticle suspensions in laboratory buffers and body fluids before removal of the salts and addition of heavy metal stain.

Known volumes of calibrant were included in methylcellulose suspension and 0.5 μl of this mixture loaded onto an EM grid. The dried circular droplets were then sampled using a systematic uniform random (SUR) pattern^45^,^46^ of imaging windows across the whole droplet. The use of SUR sampling^45^,^46^ ensures unbiasedness and optimal efficiency even with increased particle density in the droplet rim. Indeed, SUR provides better precision and is more rapid to implement than simple random sampling. The total number of gold particles in 0.5 μl was computed from the number of particles counted in the windows multiplied by the ratio of the droplet area to the collective sampled area in the windows. The concentration of gold particles in the calibration preparation used for quantifying the nanoparticles used in this study (liposomes and influenza virus) was found to be 4.4 × 10^10^ particles ml^−1^ (coefficient of error, 6.47%; n = 8; see Supplementary Figure 1).

To further validate the counting method we compared estimates of concentration in gold calibrants with an independent measure of gold particle number based on 3D models. A popular model based approach for estimating gold particle concentration assumes spherical shape of particles. However, this underestimates gold particle number because gold colloidal particles are a mix of crystalline structures such as the truncated octahedrons that are the favored for this particle size^47^. Indeed the crystalline morphology of our gold calibrant was supported by the observation of particles with multifaceted profiles and densities that were interpreted as due to electron scattering by crystal vertices. By measuring the maximal diameters of gold particles (circumscribed sphere diameters), we could then apply known ratios of sphere/crystal volumes for truncated octahedrons. We estimated a particle concentration of 1.61 × 10^12^ per ml. By comparison the droplet counting method from the same gold colloid produced a mean value of 1.688 × 10^12^ per ml (coefficient of error, 2.93%; n = 3; see supplementary materials for details). It is important to note that there are few other methods for reliable counting of small nanoparticles. We attempted to count the gold particles or liposomes using flow cytometry and nanoparticle-tracking analysis but were unable to produce consistent results.

### Quantifying nanoparticles–gold calibration/nanoparticle ratio

**Figure 5:**
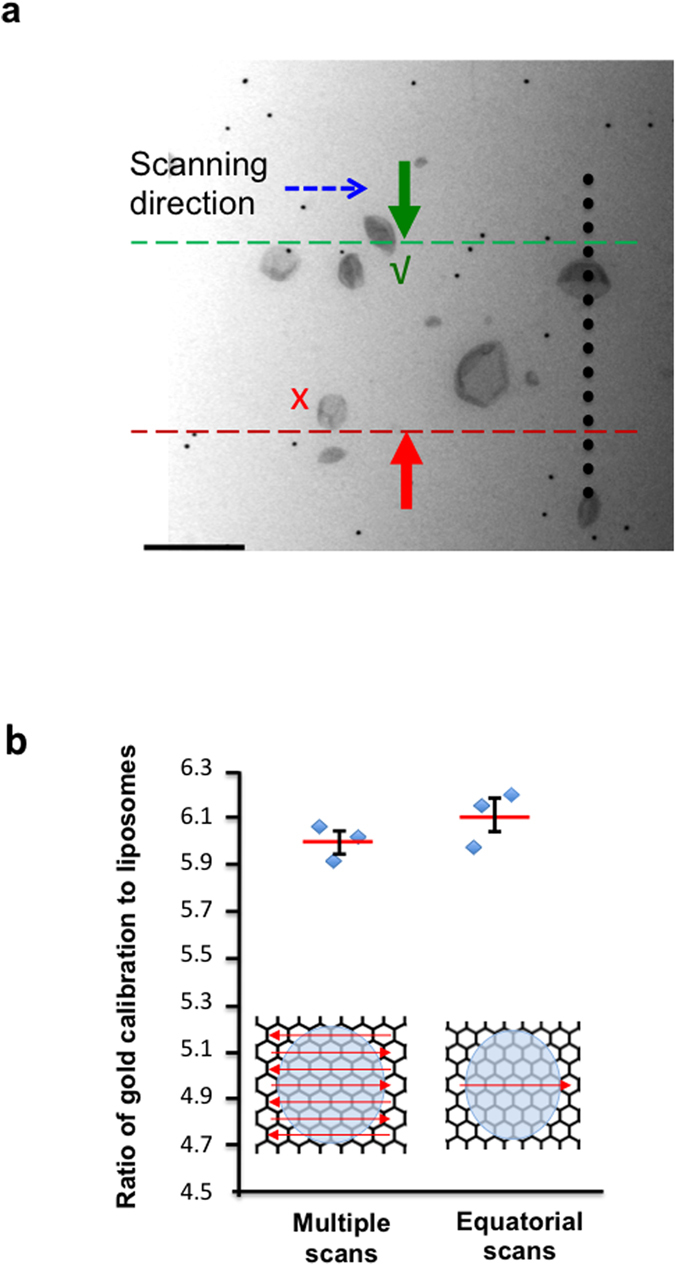
Quantification of nanoparticles using gold particle calibration. (**a**) During scanning gold particles and test nanoparticles were imaged “live” using a digital camera and counted using a scanning band as described in the text and in Fig. 4. The tip of the green arrow traces the acceptance line (dashed green) and red arrow the forbidden line (dashed red) during translocation of the specimen. The liposome (nanoparticle) concentration was estimated from the ratio of golds to liposome (nanoparticle) multiplied by the gold calibrant concentration. (**b**) Estimates of gold/nanoparticle ratio from single equatorial scans closely match those obtained from multiple SUR scans. Number estimates were obtained from the same grid but in different locations for each type of sample (n = 3, mean value and bars standard error of mean). Scale bar, 500 nm.

The use of the gold particle calibrant for counting liposomes by ratio estimation is illustrated in Fig. 5. Liposomes were mixed with methylcellulose/gold calibration solution and then uranyl acetate added (dialysis was not necessary here because the liposome carrier was compatible with heavy metal staining). 0.5 μl was loaded onto support grids before air-drying and neither liposomes nor gold particles showed evidence of aggregation. Also they did not interact substantially with each other (see Fig. 5a; <1% of gold particles were located at liposomes) and did not appear to segregate from each other in different areas of the droplet.

Gold and liposomes (nanoparticles) were counted on a live digital camera display to increase throughput (Fig. 5a). The particles were sampled by applying unbiased counting rules^45^,^46^ to a scanning band (Figs 4 and 5a) and all sampled gold and liposomes particles “exiting” the band were counted (Fig. 5b). Initially approximately 10 scans were positioned SUR across the whole droplet (SUR sampling is by definition unbiased). As already mentioned SUR is more efficient than random sampling especially when distributions are heterogeneous as is expected in the coffee ring effect^31^,^32^. However to improve efficiency further, we tested whether a single scan placed across the grid equator, would provide results that closely match the results obtained using this SUR approach; again counting approximately 100 or more gold or nanoparticles (i.e. liposomes; Fig. 5b). A single scan was much quicker to carry out and took approximately 10 minutes providing a mean estimate that was within 1.2% of the SUR value. Initially when comparing SUR scans with a single equatorial scan we counted an average of 364 gold particles and 60 liposomes per experiment. However it is a general principle in stereology that 100–200 events are usually sufficient for workable precision and if one particle type is more abundant than the other the counts can be balanced by reducing the scanning bandwidth for the most abundant particle type (the overall quantity of counts can also be altered by adjusting the magnification). One possibility is that the different particle types are distributed differently during drying especially in the droplet periphery^48^,^49^. Theoretically this effect could be detected by differences in the estimates using SUR and equatorial sampling because equatorial sampling favors the more central populations while SUR does not. Importantly the strong similarities in results from these different sampling strategies indicate that separation effects are minor or absent. It is possible that methylcellulose could reduce such separation effects by reducing the surface tension^49^.

**Figure 6:**
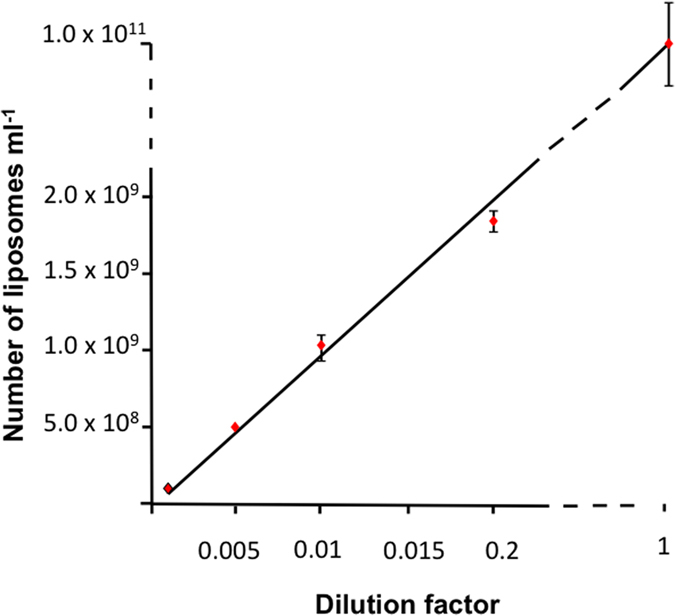
Quantification of liposomes. Scaling between liposome dilution and estimates of liposome particle number. In each case, approximately 100 each of gold calibration and liposomes were counted (time for each scan 10–15 minutes). Values are means of 4 separate dilution series derived from the same liposome preparation and error bars are coefficients of variance.

We next tested precision of number estimates across a range of dilutions. The dilutions covered approximately 4 orders of magnitude and the average coefficient of variation was 6.3%, although at the lowest concentrations the precision ranged from 1.9 to 4.1% (Fig. 6). Scaling was close to linear (Correlation coefficient R^2^ = 1). When the liposome concentration was above 10^7^/ml counting took approximately 10–15 minutes. To test the limits of sensitivity we scanned the droplet in the rim only where the film was thickest and presented the highest concentration of nanoparticles. On this basis we found an estimable concentration of nanoparticles to be approximately 5 × 10^6 ^ml^−1^.

### Quantifying a test sample–influenza A virus

**Figure 7:**
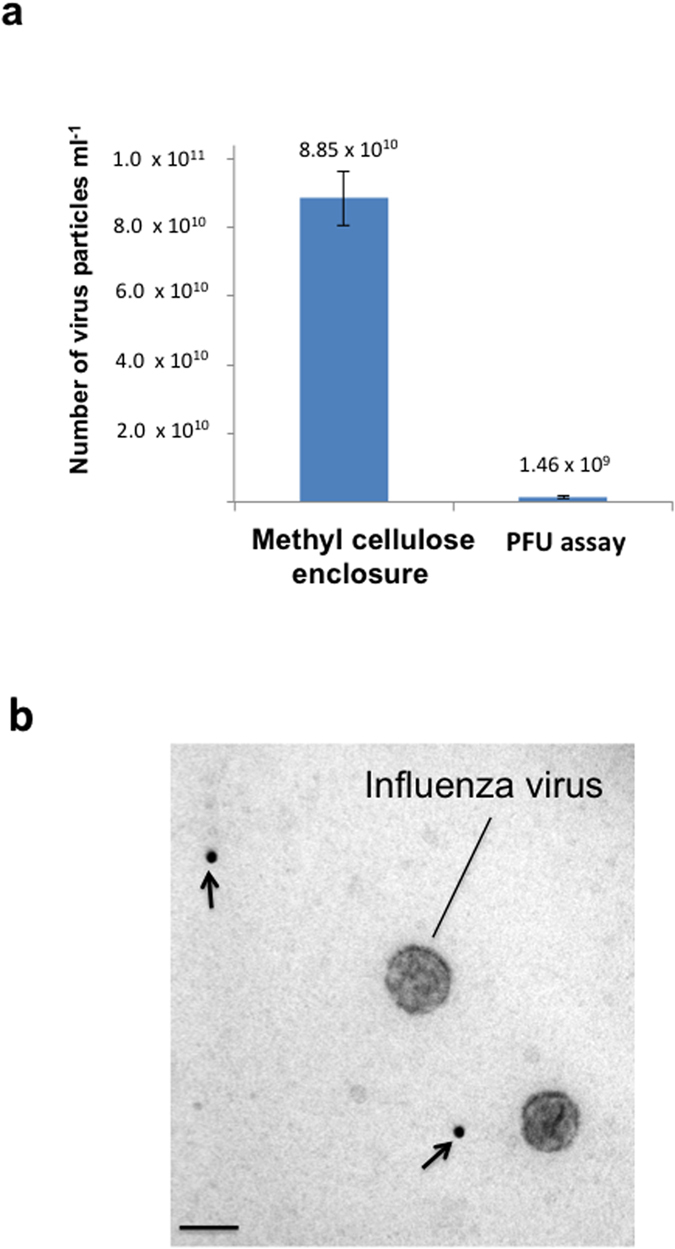
Influenza A virus quantification. (**a**) The virus preparation was mixed with gold/methylcellulose calibration solution, dialyzed by the microdialysis method (see Fig. 3) and embedded in methylcellulose/uranyl acetate. Estimates of virus particle number using the suspensions in methylcellulose method were compared with plaque forming units (PFU assay), determined on the same samples (n = 3; error bars, standard error of the mean). (**b**) TEM image showing lack of gold (arrows) or virus particle interaction/aggregation. Scale bar = 100 nm.

To test the feasibility of the counting method we estimated nanoparticle numbers in three independent preparations of influenza A virus particles. These preparations contained phosphate buffered saline and were mixed with first methylcellulose/gold calibrant before dialysis against deionized water. Uranyl acetate was then added before droplet loading and drying. Ratio counting of approximately 100 gold and 100 virus particles was carried out using single equatorial scans (Fig. 7; each count required between 5 and 10 minutes). Infectivity was also assayed in parallel using plaque forming unit (PFU) assays, allowing the particle to infectivity ratio to be determined. The particle/infectivity ratio was estimated to be 69.9:1 (coefficient of variation for particle number was 15.4% and for PFU was 47.5%; demonstrating enhanced precision of methyl cellulose embedment over the PFU method). Methylcellulose embedment proved to be much more precise and rapid than the widely used haemagglutination (HA) assay^50^ which is indirect, well known to be imprecise and relies on assumptions about thresholds of particle aggregation^51^. Our estimates of virus particle number indicate the ratio of particles to infective units might be substantially higher than indicated by previous estimates^52^.

### Workflow summary

**Figure 8:**
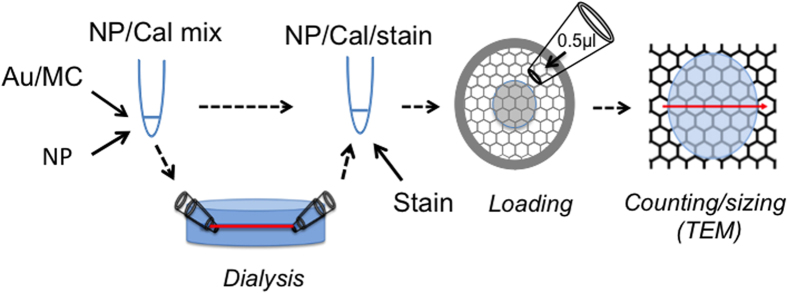
Overview of suspension in methylcellulose method. Nanoparticle (NP) suspension is added to gold calibrant (Au)/methylcellulose (MC) to make a calibration mix (NP/Cal mix). If necessary, NP/Cal mix is dialyzed in microdialysis straws and extruded before adding heavy metal stain (uranyl acetate). 0.5 μl is loaded onto the plastic coated TEM support grid for counting, sizing and further characterization.

Figure 8 summarizes the workflow for quantifying an unknown nanoparticle preparation. First the preparation is mixed 1:1 with gold/methylcellulose calibration. If necessary microdialysis of 3–5 μl of the nanoparticle/calibration mix against uranyl acetate-compatible solutions can be performed in cellulose straws. Uranyl acetate is then added to a final concentration of 0.03%, and 0.5 μl is then loaded onto a pioloform-coated EM grid before air-drying. At the TEM, a single equatorial scan across the center of the droplet is used to size nanoparticles or estimate the ratio of calibrant particles/nanoparticle according to Figs 4 and 5. The width of particle sampling bands and, if required magnification, are adjusted according to the abundance of nanoparticles/calibration gold to count 100–200 of each (the counts in the smaller band are multiplied by the ratio of the large band size to the small band size, to allow comparison).

## Conclusions

In conclusion we have developed a rapid method for counting and sizing nanoparticles at high resolution, improving on widely used biophysical methods. We consider the high precision and efficiency to be built on two main advantages. The first is the property of methylcellulose to maintain nanoparticles in homogeneous suspension, reducing aggregation and artifacts while presenting the whole population for quantitative analysis. The second is the use of rigorous and efficient sampling tools for quantitation that reduce the workload. In future further improvements in efficiency could be made by using automated feature recognition to remove reliance on operator-based counting/sizing. We recently developed algorithms for identifying and sizing particles automatically^53^ and their use in combination with the present method would improve throughput. Another improvement would be the labeling of molecular nanoparticle components, which is a powerful method for categorizing nanoparticles and membrane bound vesicles^54^. We have not extended the current technique in this way but affinity labeling techniques using multiple sizes or shapes of electron dense markers^55^ would provide correlation of size or detailed structure with molecular composition. Compared to particle tracking analysis, the method improves sensitivity by two orders of magnitude and reduces sample size from 100 s to just a few microliters. By incorporating microdialysis, microliter samples from culture supernatants or body fluids can be analyzed. Preparation, counts or size distributions take a few minutes and require little training, and use standard electron microscopy to provide nanometer resolution of structural features. Although our results show that the current method is useful for counting and sizing of nanoparticles they do not at present detect the aggregation state of the particles before they were mixed with MC. Given that aggregation state is a key parameter in nanoparticle research it will be of interest to assess the effects by independent measures of particles aggregation such as cryo-EM or DLS^18^.

The method appears to be suitable to a wide range of nanoparticle types from those composed of condensed materials with inherent electron contrast (metallic colloids or plastic beads), to those composed of soft biological materials for which electron dense staining may then be a prerequisite. The method was tested for use on nanoparticles dispersed in aqueous media, which were made compatible with the use of electron stains by prior dialysis. Future studies will reveal whether other embedding media or solvents can be used, opening up the droplet method to a wider range of nanoparticle types.

Embeddment in MC droplets should now facilitate direct analysis of tiny samples of synthetic and naturally occurring nanoparticles widely used in laboratory studies, cosmetics, pharmacology, theranostics and nanotoxicology. Specifically these applications could include: (1) counting and sizing of commercial metal colloids and plastic/polymer beads in quality control and monitoring during synthesis and use, (2) the characterization of liposomes used in drug delivery and cosmetics, (3) preparation of multifunctional nanoparticle preparations in therapeutics, (4) diagnosis of viruses and preparation of viruses in vaccine development as well as (5) the study of extracellular vesicles such as exosomes in laboratory samples and body fluids, thereby facilitating their use as markers in human disease.

## Methods

### Cells, Virus and Plaque Assays

MDCK and 293T cells were maintained in Dulbecco’s modified Eagle’s medium (DMEM, supplemented with 10% fetal calf serum; Invitrogen, Paisley, UK) at 37 °C in a 5% CO_2_ atmosphere. Influenza A virus/WSN/33 wild-type (rWSN wt) was generated using plasmid-based reverse genetics as previously described^56^. Briefly, 293T cells were transfected with eight virus genome-encoding plasmids (pHH-21-based; encoding genes under the control of the human RNA polymerase I promoter) and four protein expression plasmids encoding the genes for the viral polymerase subunits (PB1, PB2, PA) as well as nucleoprotein (nanoparticle) under control of a CMV polymerase II promoter. At 16 h post-transfection, cells were co-cultured with MDCK cells in serum-free DMEM containing 2.5 μg/mL N-acetyl trypsin (Sigma Aldrich, Dorset, UK). Virus-containing supernatant was harvested three days post-transfection, viruses propagated twice through MDCK cells, followed by plaque assay titration on MDCK cells. MDCK cells in six-well plates were infected with serial 10-fold dilutions of each virus in serum-free DMEM for 1 h at 37 °C. Cells were overlaid with DMEM–1% agarose supplemented with 2 μg/ml N-acetyl trypsin and incubated at 37 °C for 48 h. Cells were fixed in 5% formaldehyde for 1 h at room temperature. Plaques were visualized by crystal violet staining. Adeno-associated virus was prepared according to McClure *et al.*^57^.

### Reagents

2% w/v methylcellulose (25 centipoise, Sigma Aldrich, Dorset, UK) was prepared as described in Griffiths *et al.*^27^. Gold particles were prepared by citrate reduction according to Frens^44^ and stored at 4 °C. To make the calibration solution, freshly made colloid was mixed 1:1 with 2% methylcellulose. To remove citrate and sodium ions, the mix was centrifuged for 2 hours at 8000 × g at 4 °C and sedimented particles resuspended in fresh 1% methylcellulose. The calibration solution was stored at months at 4 °C. Nanodiscs were prepared as described^35^, and liposomes according to Marius *et al.*^41^. Standard plastic support films were made using 1% pioloform (Agar Scientific, Stansted, Essex, UK) and copper hexagonal 100-mesh or square 200 mesh grids applied to the film. Nanorods (10 nm thick) were obtained from Sigma Aldrich (Dorset, UK).

### Stain mixes and application to EM grids

Basic nanoparticle staining mixture (without incorporating gold calibration or pre-dialysis) contained 1.5 μl of 1% methylcellulose mixed with 7.5 μl of nanoparticle solution and 1 μl of 0.3% uranyl acetate (values are given in μl to illustrate minimal volumes but larger volumes were also used in proportion; final uranyl acetate and methylcellulose concentrations: 0.03% and 0.15% respectively). When calibration was required, 1.5 volumes of gold particle-calibration solution (containing 1% methylcellulose) were added to 1.5 volumes of nanoparticle solution, 1 volume of 0.3% uranyl acetate and 6 volumes of deionized water. When dialysis was required, equal volumes of calibration solution and nanoparticle solution were mixed and capillary dialysis carried out as detailed below. 0.5 μl of staining-mix were loaded onto pioloform-coated EM grids using a standard 2 μl micropipette under binocular control. The grids were then air-dried and examined in a JEOL 1200 EX transmission electron microscope operated at 80 kV and imaging carried out using a GATAN Orius 200 digital camera (GATAN, Abingdon, Oxon, UK).

### Capillary tube dialysis

Renal hemodialysis cassettes contain numerous parallel capillary straws that were best extracted by pulling groups from the bundle, using forceps. Immediately after drawing them out from the cassette, each straw was cut with a clean scalpel at each end. Then each end was passed through the narrower aperture of a truncated micropipette tip (as shown in Fig. 3) and the mix aspirated by capillary action. To seal the tube, another pipette tip was then inserted into the wider aperture of the first tip, thereby trapping the capillary. The whole assembly was subsequently immersed in dialyzing solution. After dialysis the tube was laid onto parafilm and curved forceps drawn along the tube to expel the dialysate. The drop was retrieved and stored in an Eppendorf centrifuge tube at 4 °C.

### Counting and Sizing procedures

These procedures were carried out directly on a digital camera display (see Figs 4 and 5). Counting was done by manual translocation of the specimen in a single direction and particles selected using unbiased counting rules applied to the edges of a counting band. The edges of the band were defined by lines traced out by two arrows positioned inside opposite sides of the image frame, thereby creating a peripheral guard area. This allowed morphological characterization/size estimation even of the largest nanoparticles. During scanning one edge of one of the spots defined an acceptance line and the edge of the other spot defined a forbidden line. Any particle that fell completely within the lines, or was crossed by the acceptance line, was sampled/accepted for counting. Any particle that intersected the forbidden line was not sampled or counted^42^,^43^.

To evaluate the influence of methylcellulose on the binding of nanoparticles to the support film, liposomes were mixed 1:1 with 2% methylcellulose or deionized water and the mix allowed to adsorb onto an EM grid for 2 minutes. After three washes on droplets of deionized water (2 minutes per step), followed by contrasting with 3% (w/v) uranyl acetate/2% methylcellulose (ratio of 1:9); grids were air-dried in a wire loop (according to Griffiths *et al.*^27^). The number of liposomes per unit area was assessed in micrographs taken SUR at 2,000×. Counting of liposomes was carried out in rectangular quadrats measuring 2.1 μm × 2.1 μm to which unbiased counting rules were applied^43^ and counts related to the area.

For estimating particle caliper diameter, a systematic series of dots with regular spacing was placed orthogonal to the travel direction of the microscope stage. The spacing was set to about half the size of the smallest nanoparticle and the extent of the array was greater than the width of the counting band so that any peripheral particles sampled by the counting band were sized. Consistently, one extreme edge of each dot was assigned for counting intersections and the number of edges that crossed each particle during translocation of the specimen was counted. An unbiased estimate of the particle caliper distance is ∑I × d where I is the number of intersections and d the real spacing between the dots.

To compare a random point process to the distribution of calibration nanoparticles, 20 micrographs, positioned systematically with a random start (SUR), were taken for each nanoparticle (magnification 3000 x for liposomes and 4000 x for influenza A virus). The images were opened in Photoshop CS6 and gold particle counts made using the unbiased counting rule described above (quadrat areas 9.49 μm^2^ for liposomes and 6.15 μm^2^ for influenza A virus). The gold calibration particles were categorized as “associated” or “non-associated” with nanoparticles (particles less than one particle width distant from the nanoparticle were classified as associated). For the distribution of random points the same micrographs were overlaid with a randomly placed square systematic grid lattice (spacing 2.05 μm for liposomes and 1.55 μm influenza A virus) resulting in 1 to 4 random points per placement. The random points were categorized as for the calibration particles. The process of random grid placement was repeated until the number of random points matched gold particles for the corresponding micrograph.

To assess the relationship between estimated nanoparticle number and dilution, a dilution series of liposomes was prepared in deionized water using 10 μl of neat liposome solution for every dilution (1/50, 1/100, 1/200 and 1/1000). For each sample four staining mixes were prepared and 0.5 μl per mix loaded onto individual EM grids. The grids were then quantified and the liposome number for each sample estimated (see above) and plotted in Microsoft Excel.

## Additional Information

**How to cite this article**: Hacker, C. *et al.* Nanoparticle suspensions enclosed in methylcellulose: a new approach for quantifying nanoparticles in transmission electron microscopy. *Sci. Rep.* **6**, 25275; doi: 10.1038/srep25275 (2016).

## Supplementary Material

Supplementary Information
