# Supplementary material for: Nanoparticle suspensions enclosed in methylcellulose: a new approach for quantifying nanoparticles in transmission electron microscopy
Source: Sci Rep. 2016 May 4;6:25275. doi: 10.1038/srep25275 (PMC4855187; doi:10.1038/srep25275)
Supplement: Supplementary Information [file srep25275-s1.pdf]

Nanoparticle suspensions enclosed in methylcellulose: a new approach for quantifying nanoparticles in transmission electron microscopy

Christian Hacker[a], Jalal Asadi[a], Christos Pliotas[c], Sophie Ferguson[a], Lee Sherry[b], Phedra Marius[c], Javier Tello[a], David Jackson[b], James Naismith[c] and John Milton Lucocq[a]

[a]School of Medicine, University of St Andrews, St Andrews, Fife, UK.

[b]Biomedical Sciences Research Complex, University of St Andrews, North Haugh, St Andrews, United Kingdom.

[c]Centre for Biomolecular Sciences, University of St. Andrews, St. Andrews, Scotland.

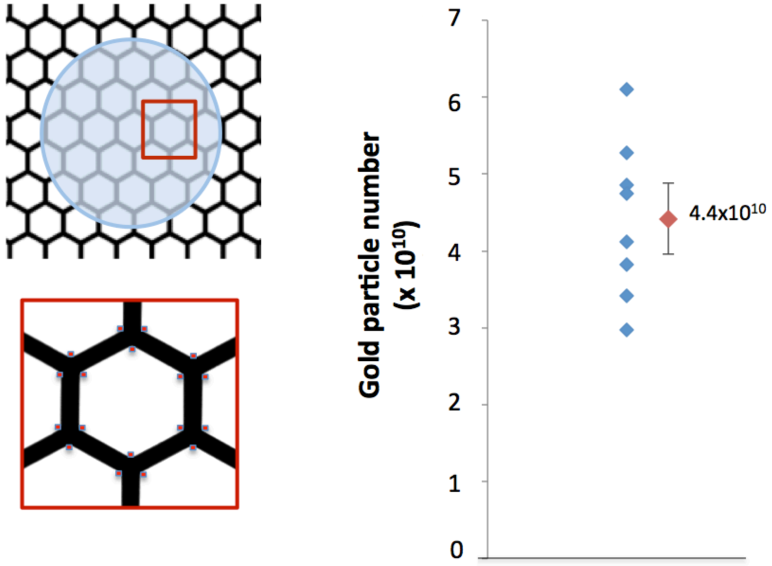

Supplementary Figure 1: Quantification of gold calibration. Quantification of gold calibrant using SUR sampling of MC droplets loaded onto hexagonal EM support grids. Images were taken at all the grid "corners" situated over the droplet. The expanded view (red square) shows the pattern of sampled images taken at the location of each red dot. Gold particles were counted by applying unbiased counting rules to the quadrats used for counting (not shown)[42,43]. The total area of the droplet was calculated by counting the number of grid corners that land over the droplet multiplied by 1/6 of the hexagonal tile area. An estimate of the total particles per droplet in a volume of 0.5 μl was the ratio of the total area of the droplet to the sampled area of the quadrats used for counting multiplied by the gold particles counted. Results for this particular gold calibrant were used for quantifying liposomes and influenza virus.

Supplementary materials and methods

15 nm diameter gold particles made using the citrate reduction method of Frens[44] were stored in 1 % MC and droplets applied to pioloform EM support grid films. Total numbers of gold

particles per milliliter were estimated by systematic sampling[45,46] at the hexagon grid corners as shown. The total number of gold particles is estimated by multiplying the sampled gold particles with the ratio of total droplet area to sampled area (both estimated using point sampling stereology). Each data point is from a separate loading experiment (n = 8, error bar standard error of the mean).

To validate the counting method the number of gold particles counted in a colloidal gold calibrant preparation was compared to that calculated using models of particle shape. Gold calibrant was prepared as described in the materials and methods and the dilution from the parent sol determined using spectrophotometry at a wavelength of 525 nm[34]. Three 0.5 μl droplet preparations were then evaluated using SUR sampling on square 200 mesh grids. In this case we increased the intensity of SUR sampling in grid squares containing the rim (thereby dramatically decreasing the inter-grid variation in the estimates and reducing overall work load). The results from the three grids were obtained were 1.77954×10^{12} 1.60957×10^{12} and 1.67606×10^{12}; mean value 1.68839×10^{12} (coefficient of error 2.93 %).

Commonly applied model based estimations of gold colloid concentration use the gold particle diameter and assume the particles are spherical. However at high power in TEM we confirmed the 15 nm particles made by citrate reduction display a variety of morphologies with corners, flat edges and internal densities typical of crystalline structures. Colloidal gold, in the 3-100 nm range, is proposed to be composed of truncated octahedral crystals[47], crystals that have volumes substantially smaller than their cognate circumscribed spheres. To improve the model-based estimates, we first estimated the volume of circumscribed spheres by measuring the

maximal caliper diameter of the gold particles (16.48314127; coefficient of error 0.102 % n = 88). We then corrected the volume estimates using the volume fraction occupied by truncated octahedrons (0.6833). Knowing the weight of gold in the colloidal synthesis reaction we calculated the particle number to approximate to 1.61 x 10^{12}, which is close to that obtained using the counting method.
